# Minimal Evidence for a Secondary Loss of Strength After an Acute Muscle Injury: A Systematic Review and Meta-Analysis

**DOI:** 10.1007/s40279-016-0528-7

**Published:** 2016-04-21

**Authors:** Gordon L. Warren, Jarrod A. Call, Amy K. Farthing, Bemene Baadom-Piaro

**Affiliations:** 1 Department of Physical Therapy, Byrdine F. Lewis School of Nursing and Health Professions, Georgia State University, PO Box 4019, Atlanta, GA 30302 USA; 2 Department of Kinesiology, University of Georgia, Athens, GA USA; 3 Regenerative Bioscience Center, University of Georgia, Athens, GA USA; 4 School of Public Health, Georgia State University, Atlanta, GA USA

## Abstract

**Background:**

An immediate loss of strength follows virtually all types of muscle injury but there is debate whether the initial strength loss is maximal or if a secondary loss of strength occurs during the first 3 days post-injury.

**Objective:**

The objective of this analysis was to conduct a systematic review and meta-analysis of the research literature to determine if a secondary loss of strength occurs after an injurious initiating event.

**Methods:**

Literature searches were performed using eight electronic databases (e.g., PubMed, Cochrane Library). Search terms included skeletal muscle AND (injur* OR damage*) AND (strength OR force OR torque). The extracted strength data were converted to a standard format by calculating the standardized mean difference, which is reported as the effect size (ES) along with its 95 % confidence interval (CI). The calculation of ES was designed so that a negative ES that was statistically less than zero would be interpreted as indicating a secondary loss of strength.

**Results:**

A total of 223 studies with over 4000 human and animal subjects yielded data on 262 independent groups and a total of 936 separate ESs. Our overall meta-analysis yielded a small-to-medium, positive overall ES that was statistically greater than zero (overall ES = +0.34, 95 % CI 0.27–0.40; *P* < 0.00000001). Considerable variation in ES was observed among studies (*I* ^2^ = 86 %), which could be partially explained by the research group conducting the study, sex of the subject, day of post-injury strength assessment, whether fatigue was present immediately post-injury, and the muscle group injured. From the subgroup meta-analyses probing these variables, 36 subgroup ESs were calculated and none were statistically less than zero.

**Conclusion:**

Overall, our findings do not support the presence of a secondary loss of strength following an acute muscle injury, and strongly suggest that strength, on average, recovers steadily over the first 3 days post-injury.

**Electronic supplementary material:**

The online version of this article (doi:10.1007/s40279-016-0528-7) contains supplementary material, which is available to authorized users.

## Key Points

**Table:** 

On average, strength does not deteriorate in the first 3 days after a muscle injury.
Care should be taken when debating the use of therapeutic interventions designed to prevent or attenuate a strength loss associated with secondary muscle injury.

## Introduction

Injury to skeletal muscle induced by work, exercise, and most traumatic means (e.g., crush, laceration, penetration, blast, freezing) results in an immediate loss of strength. Depending on the means for inducing injury, this strength loss can be attributed to a disruption of the excitation–contraction coupling process and/or frank damage to force-generating or -transmitting structures within the muscle [1–3]. We and others have hypothesized that the initial injury can start a cascade of events that leads to additional injury in the ensuing hours and days [3, 4]. This cascade has been thought to begin with a loss of intracellular calcium homeostasis within the damaged muscle fibers that is brought on by a loss of plasmalemmal integrity. The loss of calcium homeostasis may then lead to activation of several degradative pathways intrinsic to muscle that are referred to as autogenetic mechanisms [4]. These calcium-sensitive pathways include calcium-activated neutral proteases and those of the phospholipase A_2_ cascade, which produce arachidonic acid, prostaglandins, and leukotrienes that may further damage cell membranes. Elevated intracellular calcium may also disrupt mitochondrial respiration and result in sarcomeric contracture. The autogenetic mechanisms for inducing damage are thought to be followed by an inflammatory phase, beginning 2–6 h after the initiating event [4]. In this phase, neutrophils and macrophages invade the damaged tissue and are primarily responsible for removal of that tissue over the next several days. It is believed that this inflammatory response may induce additional injury by spillover of inflammation, including reactive oxygen species, from damaged tissue onto adjacent tissue that was initially undamaged (i.e., the so-called bystander injury) [5].

If the autogenetic mechanisms and inflammatory response contribute to an additional (or secondary) injury in the hours and days following the initiating event, the damage should be measurable using standard markers of muscle injury. Such markers include histopathology, soreness, blood levels of muscle proteins, limb range of motion, and strength. We and others have reasoned that muscle strength, measured during either maximal voluntary or electrically elicited contractions, provides the single-best assessment of the extent of muscle injury regardless of injury type. This is because strength comes closest to evaluating the overall functional capacity of the tissue [3, 6]. Strength is also quantifiable in both human and animal injury models whereas many other measures are not feasible or practical in all models (e.g., quantitative histopathology in human models) and/or are only semi-quantitative (e.g., blood levels of muscle proteins).

The earliest suggestion for a secondary loss of strength following an initiating injurious event came from John Faulkner and colleagues in a narrative review article published over 20 years ago [3]. They specifically argued that a secondary strength loss occurs in the first 3 days after injury. Since then, others have acknowledged that such a strength loss is likely [7, 8], but the direct evidence for the loss has been mixed (e.g., Brooks and Faulkner [9] and Roche et al. [10]). It is important to determine whether a secondary strength loss occurs because this information can affect if and how muscle injuries should be treated, specifically whether interventions such as anti-inflammatory medications, cryotherapy, or antioxidants should be used to block autogenetic pathways or minimize the inflammatory response. It is possible that these cellular events are more associated with repair and regeneration of the damaged tissue than with inducing an additional (or secondary) injury. If this is the case, the above interventions may have no beneficial effects at best and adverse effects at worst.

**Fig. 1:**
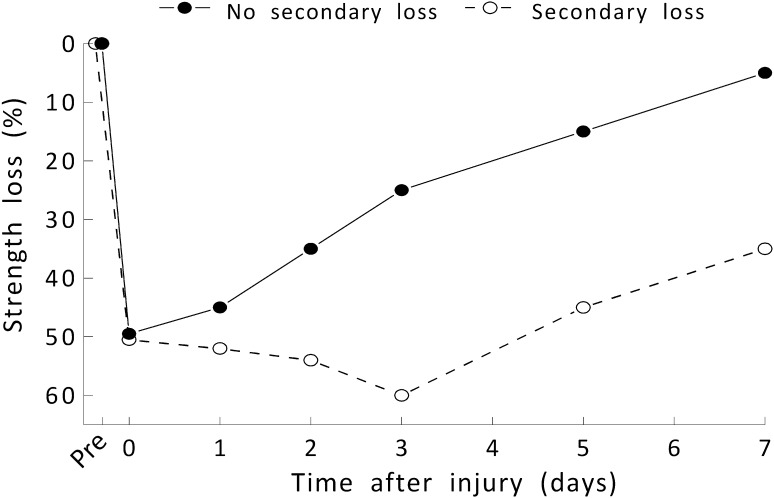
Hypothetical graph of strength loss vs. time post-injury with and without a secondary loss of strength

Because of the apparent discrepant evidence in the literature for the presence of a secondary strength loss, we felt that a rigorous, quantitative analysis of the literature was warranted. Our objective was to conduct a systematic review combined with a meta-analytic approach to determine whether over the first 3 days following an injurious event to muscle, there is a strength loss above and beyond the initial loss (Fig. 1). Such a loss would constitute a secondary strength loss and a secondary muscle injury. We are unaware of any previous attempt to address this issue using this methodology. We also sought to explain the disparate findings in the literature by examining the effects of various experimental factors (e.g., subject type [animal vs. human], sex of the subject, type of injury, muscle group injured, day of post-injury strength assessment) that have varied among studies.

## Methods

### Systematic Review

#### Selection of Studies

A thorough systematic search of the research literature was performed conforming to the PRISMA statement [11] to determine if a secondary loss of strength occurs over the first 3 days after a muscle injury. Our literature search began in January 2011 and continued through to December 2012. The databases that were searched include PubMed, SPORTDiscus, ISI Web of Knowledge, CINAHL, Cochrane Library, OpenSIGLE, ProQuest Dissertation and Theses, and the American College of Sports Medicine database of annual meeting proceedings. The search terms used were skeletal muscle AND (injur* OR damage*) AND (strength OR force OR torque). Reference lists from fully evaluated publications were also examined for studies not found with the online database searches.

#### Study Inclusion and Exclusion Criteria

Studies meeting the following criteria were considered for review: (1) muscle injury had to be induced experimentally by exercise or work biased to the performance of eccentric contractions or by trauma (e.g., crush, laceration, penetration, blast, freezing, myotoxin injection, ischemia–reperfusion); (2) strength in the injured muscle group had to be assessed immediately post-injury (i.e., within the first 6 h) in addition to at least one assessment performed between 24 and 72 h post-injury; and (3) isometric, isokinetic, and/or isotonic strength was assessed using maximal voluntary and/or electrically elicited contractions. Studies or study subgroups were excluded for the following reasons. First, subjects performed non-damaging exercise (e.g., isometric or concentric contractions) in addition to or in lieu of eccentric contractions or eccentric contraction-biased exercise. Second, drugs, ergogenic drink/food, or supplements were ingested or administered prior to, during, or after injury. However, if one or more independent groups of subjects from a study met the above criteria for inclusion (e.g., a control group whose muscles were injured but received sham treatment in an interventional study), their data were included in the analysis. Third, a therapeutic modality such as massage, heat, ice, transcutaneous electrical nerve stimulation, ultrasound, or hyperbaric oxygen was applied prior to, during, or after injury. Fourth, for studies inducing injury by eccentric contractions, a bout of eccentric contractions had been performed in the previous 3 months; in other words, the second bout in repeated-bout studies was excluded. Fifth, animal strains modeling a disease were used (e.g., the *mdx* mouse, which is a model for Duchenne muscular dystrophy). Sixth, if there were insufficient data reported in a study to calculate an effect size (ES) for the change of strength over the first 3 days post-injury, the study was excluded. Before excluding such studies, we attempted to retrieve the necessary data by contacting the corresponding author by e-mail and/or telephone.

**Fig. 2:**
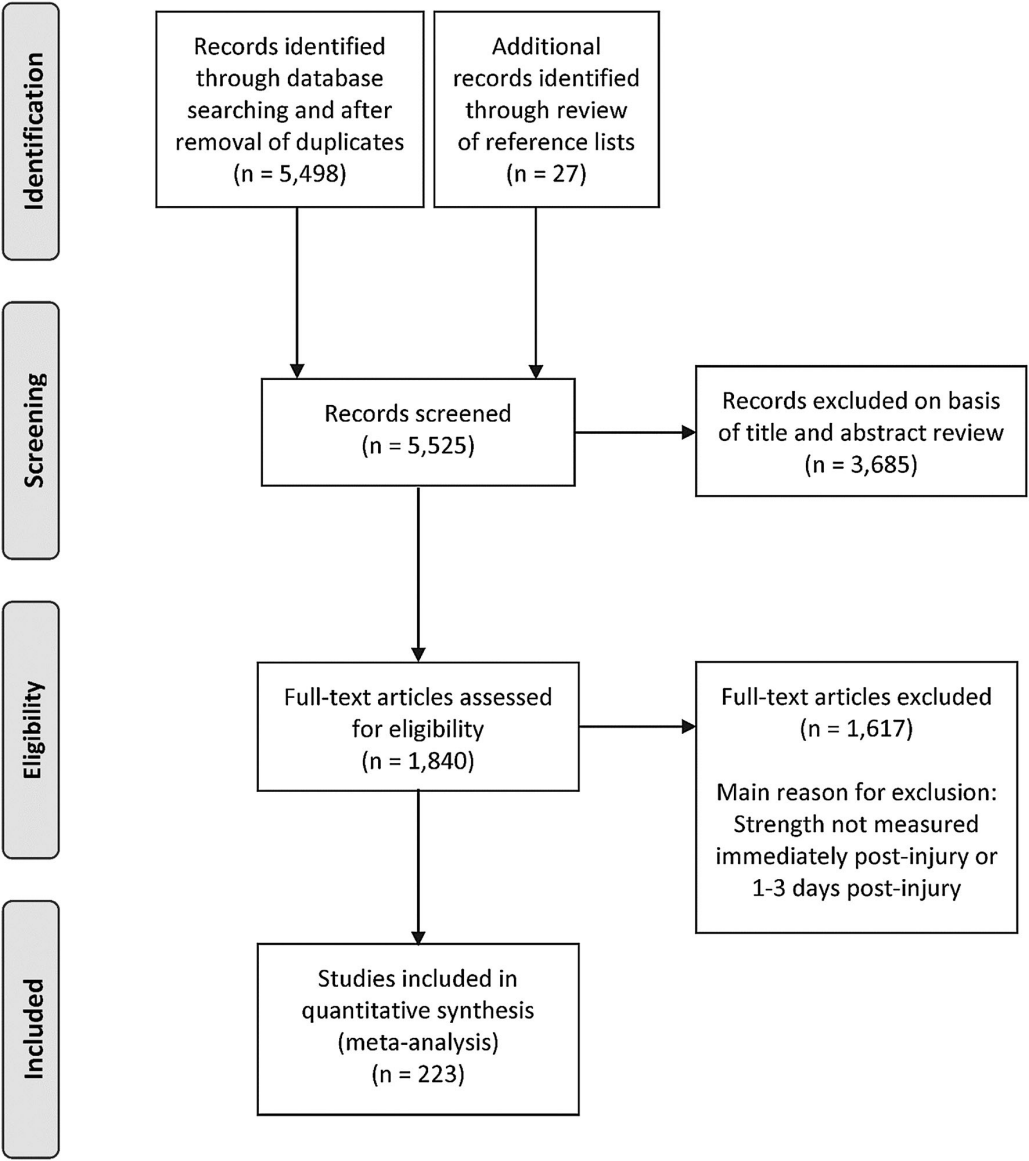
Flowchart for review and selection of studies in the systematic review

A total of 5525 non-duplicate studies were originally identified through the database searches and review of article reference lists. Of those, 3685 were initially excluded on the basis of reviewing the title and abstract. At this point, 1840 were fully evaluated via a careful review of the full-text article. Using the inclusion and exclusion criteria, 1617 studies were excluded, leaving a total of 223 studies to be included in the meta-analyses. The review and selection processes for the studies in the systematic review are summarized in Fig. 2. Each step of the review and selection processes was conducted independently by at least two of the authors. If there was disagreement among the two authors, a third author was recruited to settle the dispute.

#### Data Extraction and Assessment of Study Quality or Bias

For calculation of study ESs to be used in the meta-analysis, strength data were extracted in the form of means, sample sizes, and standard deviations (SDs) or standard errors (SEs) for all post-injury timepoints, strength measures, and subject groups meeting our criteria. In studies that did not report all three descriptors, the following were extracted: (1) means, sample sizes, and *P* value; or (2) effect direction, sample sizes, and *P* value. If available for repeated-measures design studies, individual subject data were also extracted so that between-trial correlations for the strength measures (i.e., correlation between that measured immediately and day 1–3 post-injury) could be calculated. Study quality and/or bias was not formally assessed in the 223 studies because there were no studies whose objective was to examine the existence of a secondary strength loss, which was the objective of the present analysis. Thus, if a study’s quality was poor or if study bias existed with regard to a study’s objective, this should not have a systematic influence on a study’s ES in our meta-analysis.

### Meta-Analysis

The extracted strength loss data were converted to a standard format by calculating the standardized mean difference, which will be referred to as the ES. The standardized mean difference calculation was set up so that a negative value would indicate a secondary strength loss. For studies in which the immediate and day 1–3 post-injury strength measures were taken on the same subjects and also means, SDs, and sample sizes were reported (i.e., the most common scenario; *n* = 197 studies), the paired difference (i.e., day 1–3 post-injury strength mean − immediate post-injury strength mean), paired difference SD [i.e., (“day 1–3” SD^2^ + “immediate” SD^2^ − 2 × between-trial correlation × “day1–3” SD × “immediate” SD)^½^], and paired difference SE (i.e., paired difference SD/n^½^) were initially calculated. These were then used to calculate the standardized mean difference [i.e., paired difference × (2 × (1 − between-trial correlation))^½^/paired difference SD] and standardized mean difference SE [i.e., (1/n + standardized mean difference^2^/2n)^½^ × (2 × (1 − between-trial correlation))^½^]. Because between-trial correlations could be calculated for only 14 independent groups, the median between-trial correlation (0.803) was substituted in the calculations for studies without correlations. For the studies in which independent groups of subjects were used within a study for measurements at the different timepoints (i.e., *n* = 26 studies), standardized mean differences were calculated as detailed previously [12].

When a study measured strength under multiple conditions (e.g., measured both isokinetic and isometric strength or measured strength multiple times over the first 3 days post-injury) using the same group of subjects, the standardized mean differences and variances were calculated for each condition level and then averaged across the different condition levels for the group. When a study had more than one independent group of subjects that met the criteria for assessing secondary strength loss, a standardized mean difference and variance was calculated for each group. Then, in the calculation of the overall standardized mean difference, these groups were treated as if they were independent studies [12]. Though 223 studies were used in the meta-analysis, these studies yielded 262 independent groups of subjects and a total of 936 separate ESs.

Meta-analyses were run using a random-effects model that accounts for true between-study variation in effects as well as for random error within each study. A random-effects model was chosen over a fixed-effect model because of the wide variation in experimental factors (e.g., use of humans vs. animals, different means of inducing injury) among studies. Between-study variation in true ES, or heterogeneity, was assessed by *Q* and *I* ^2^ statistics. Because heterogeneity was found to be high, meta-regressions utilizing a method-of-moments approach and subgroup meta-analyses utilizing a *Q*-test based on ANOVA were used to investigate potential moderator variables (experimental factors) as possible explanations for the heterogeneity. The potential moderator variables examined were (1) study publication year; (2) subject type (human vs. animal); (3) subject sex; (4) subject age; (5) day of post-injury assessment of muscle strength (day 1 vs. day 2 vs. day 3); (6) for human studies, the muscle group injured (e.g., elbow flexors vs. knee extensors); (7) for animal studies, the type of rodent (rat vs. mouse); (8) for animal studies, the muscle injured (ankle dorsiflexor vs. plantarflexor); (9) for animal studies, the type of injury (eccentric contractions vs. traumatic); (10) presence of fatigue immediately post-injury; (11) magnitude of the immediate strength loss; (12) type of contraction used to assess strength; and (13) research group conducting a study.

It was important to determine whether fatigue was present in a study because it might explain some of the between-study variation in ES. If fatigue was present after injurious work or exercise, the immediate post-injury strength would be lower than it should be. As a result, the study ES would be inflated and possibly positive when it should have been negative. To determine whether fatigue was present in a study, we examined the methods for each study to see if (1) there was a comparable control group (e.g., using isometric or concentric contractions as a control for injury induced by eccentric contractions); or (2) multiple strength measurements were made in the 0–6 h post-injury period so that strength changes over this period could be assessed. If the strength loss in a control group was apparent as compared with that for an eccentric contraction-injured group or if recovery of strength was apparent in the eccentric contraction-injured group over 0–6 h post-injury, fatigue was said to be present immediately post-injury in that study. For studies without these controls but in which (1) the injury was traumatic in nature (i.e., no contractions were performed during injury induction); or (2) the immediate post-injury measure of strength was performed relatively late after the eccentric contraction injury protocol (i.e., 0.5–6 h post-injury), we assumed that fatigue was not present immediately post-injury. For all other studies, the presence of fatigue immediate post-injury was listed as unknown.

To determine if the research group conducting a study might also explain some of the between-study variation in ES, we designated research groups using the following procedure. First, we identified authors of studies from our meta-analysis that had conducted five or more studies. These authors were then cross-referenced with each other to identify collaborative groups (e.g., relationships between an advisor and his/her current or former students, post-doctoral fellows, or colleagues). Each collaborative group was required to have ten or more independent groups for which an ES was calculated in the meta-analysis. We identified five research groups that met these criteria. Studies not belonging to a research group were placed in a group labeled “All other studies.”

For all subgroup meta-analyses, each subgroup was required to have a minimum of five studies, with the exception as noted in the previous paragraph. In studies that had more than one level of the experimental factor being evaluated (e.g., a study measuring strength loss in both a group of men and a group of women in the subgroup meta-analysis evaluating the effect of subject sex), an ES was calculated for each level and each ES was treated as if it originated from an independent study.

Meta-analyses, subgroup meta-analyses, and meta-regressions were performed using the Comprehensive Meta-Analysis software (version 3.3; Biostat Inc., Englewood, NJ, USA). ESs of 0.2, 0.5, and 0.8 were considered to be small, medium, and large, respectively [13]; an ES of 0.1 was considered to be trivial. An α level of 0.05 was used in all analyses except in subgroup meta-analyses where more than two levels of a moderator variable existed and the overall *Q*-test yielded a significant *P* value. In this situation, a Benjamini and Hochberg false discovery rate adjustment was applied to the α level to correct for multiple post hoc pairwise comparisons using *Q*-tests. As for study quality and bias as mentioned above, the effect of publication bias on the meta-analysis was not assessed because no studies in the meta-analysis had a research objective matching that of the present analysis. Though unpublished studies meeting our inclusion/exclusion criteria are likely, these should not be biased towards exhibiting a secondary strength loss or not.

## Results

### Description of Included Studies

In total, 223 studies, along with data for 262 independent groups of subjects, were published between 1985 and 2012, and these were included in the meta-analysis investigating secondary strength loss. Briefly, there were 184 human and 39 animal studies. For the human studies, there were a total of 3413 subjects, with the average age for a study ranging from 18 to 70.5 years. These studies contained 111 independent groups that were male only and 22 that were female only. For the human studies, strength was reported both immediately and at day 1, day 2, or day 3 post-injury in 192, 188, and 163 independent groups, respectively. Eccentric contraction-biased exercise or eccentric contractions were used to induce injury in all human studies. For the animal studies, there were a total of 664 rodents, with the average age for a study ranging from 2 to 27 months. These studies contained 24 independent groups that were male only and 11 that were female only. For the animal studies, strength was reported both immediately and at day 1, day 2, or day 3 post-injury in 14, 11, and 40 independent groups, respectively. Animal studies employed several injury models (i.e., eccentric contraction-induced injury, downhill walking/running, blunt-impact injury, ischemia–reperfusion injury, and freeze injury).

### Meta-Analysis of Secondary Strength Loss

Considerable variation in ES was observed among studies, with ES ranging from −2.46 [9] to +6.29 [14] (Electronic Supplementary Material Figure S1). As illustrated in the forest plot, 67 of the 262 independent groups exhibited a negative ES, which supports a secondary loss of strength occurring at 1–3 days post-injury. Conversely, 195 independent groups exhibited a positive ES, indicating an absence of secondary strength loss in those studies. With all independent groups included, the meta-analysis yielded a small-to-medium overall ES that was positive and statistically greater than zero (overall ES = +0.336, 95 % confidence interval [CI] 0.270–0.401; *P* < 0.00000001; Electronic Supplementary Material Figure S1), indicating that, on average, muscle strength improves over the first 3 days post-injury. As one would expect, there was no one study that dominated the overall ES. The study of Rodenburg et al. [15] had the single largest effect on the overall ES. If this study was removed from the meta-analysis, the overall ES would only fall to +0.326 and the effect would still be statistically greater than zero (*P* < 0.00000001).

The overall ES was also calculated using the most liberal scenario for possibly detecting a secondary strength loss. Because multiple ESs were calculated for most studies, we opted to run the primary meta-analysis using the single smallest (or most negative) ES determined for each independent group or study. The overall effect (ES = +0.067; 95 % CI 0.004–0.130; *P* = 0.036) when calculated this way was still positive, albeit trivial in magnitude, and was significantly greater than zero. Thus, this analysis also does not provide support for the occurrence of a secondary strength loss.

Tests of heterogeneity were performed to assess the extent of between-study variation in the ES. Because heterogeneity was large and statistically significant (*I* ^2^ = 86 %; *Q* = 1900, *P* < 0.00000001), moderator variables that could potentially explain this heterogeneity were investigated using subgroup meta-analysis and meta-regression. With the exception of one moderator variable, Table 1 summarizes the findings of the subgroup meta-analyses. Subject type was not a significant moderator variable as there was no statistical difference in ES between studies using humans and those using animals. Likewise, within animal studies, there was no significant difference in ES between studies using rats and those using mice. The sex of the subject was, however, able to explain a significant portion of the heterogeneity (*P* = 0.02). Interestingly, the ES determined for studies using males was more than twice that for studies using females, suggesting that males may recover strength faster after injury.

**Table 1:** Summary of subgroup meta-analyses examining nominal moderator variables that might explain between-study variance in effect size

Moderator variable	Comparison^a^	*Q*-test *P* value
Subject type	Animal (*n* = 46, ES = +0.36 [0.18–0.54]) vs. human (*n* = 216, ES = +0.33 [0.26–0.40])	0.80
Rodent type	Mice (*n* = 32, ES = +0.26 [0.01–0.51]) vs. rat (*n* = 13, ES = +0.77 [0.32–1.21])	0.05
Subject sex	Female (*n* = 33, ES = +0.17 [–0.04 to 0.37]) vs. male (*n* = 135, ES = +0.45 [0.35–0.55])	0.02
Day of post-injury assessment	Day 1 (*n* = 206, ES = +0.19 [0.12–0.27]) vs. day 2 (*n* = 199, ES = +0.34 [0.27–0.42]) vs. day 3 (*n* = 203, ES = +0.52 [0.44–0.59])	0.00000004 Day 3 > day 2 > day 1
Muscle group (human studies)	Elbow flexors (*n* = 117, ES = +0.46 [0.37–0.54]) vs. knee extensors (*n* = 81, ES = +0.11 [–0.00 to 0.22]) vs. knee flexors (*n* = 11, ES = +0.14 [–0.16 to 0.44])	0.000007 Elbow flexors > knee extensors, elbow flexors > knee flexors
Muscle group (animal studies)	Ankle dorsiflexor (*n* = 40, ES = +0.33 [0.09–0.56]) vs. ankle plantarflexor (*n* = 7, ES = +0.98 [0.40–1.56])	0.04
Injury type (animal studies)	Eccentric contraction-induced injury (*n* = 41, ES = +0.40 [0.17–0.64]) vs. traumatic injury (*n* = 5, ES = +0.31 [–0.32 to 0.94])	0.78
Presence of fatigue immediately post-injury?	Yes (*n* = 27, ES = +0.62 [0.39–0.85]) vs. no or not likely (*n* = 89, ES = +0.32 [0.19–0.46]) vs. unknown (*n* = 146, ES = +0.30 [0.22–0.38])	0.03 Yes > unknown
Type of contraction used to assess strength	Isometric (*n* = 234, ES = +0.33 [0.26–0.40] vs. isokinetic or isotonic (*n* = 44, ES = +0.30 [0.14–0.45]	0.67

*ES* effect size

^a^Sample size (*n*) refers to the number of independent groups of subjects in a subgroup. Values within brackets represent the 95 % confidence interval for the ES. Analyses were run on data for human and animal studies combined except where noted otherwise

**Fig. 3:**
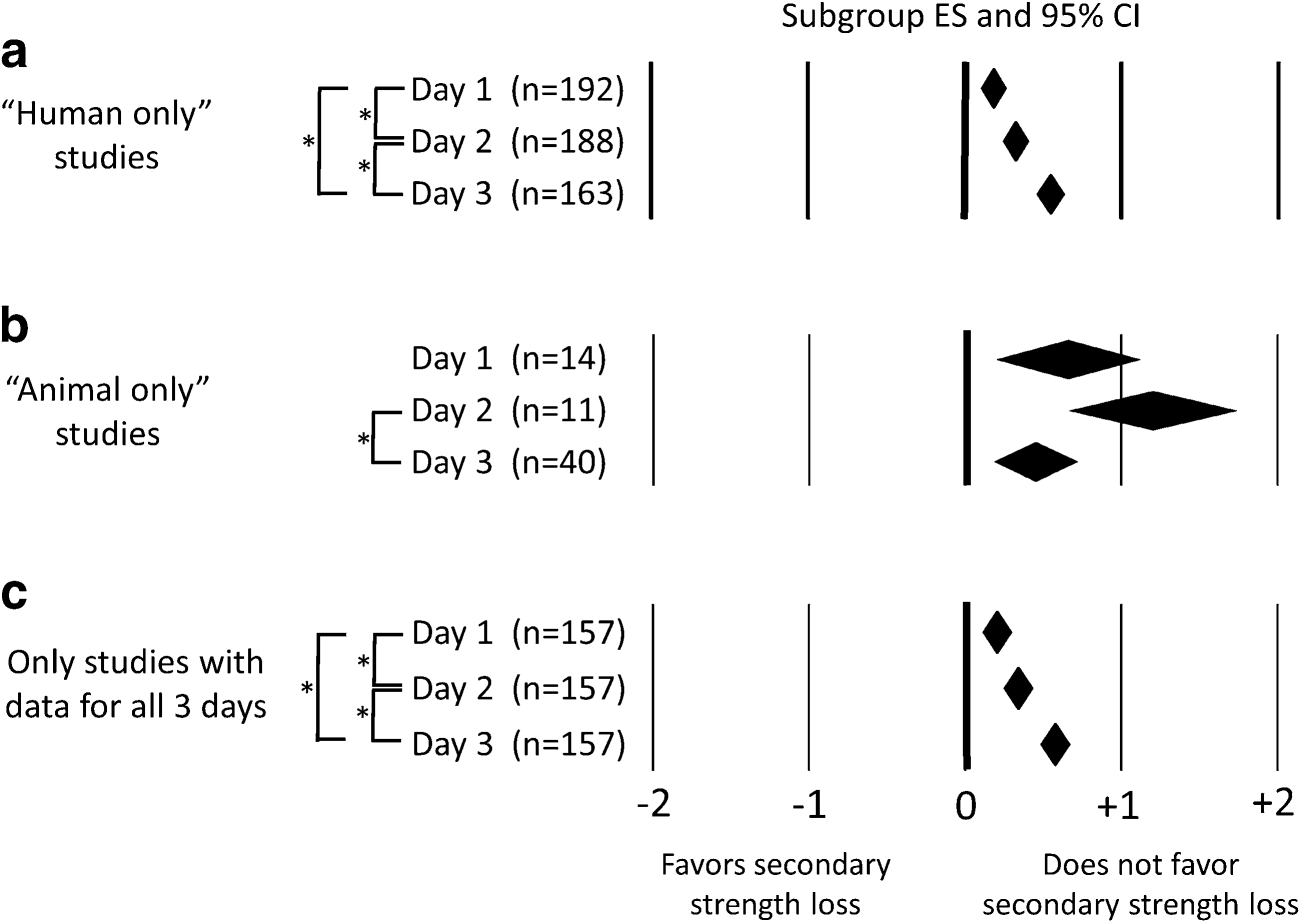
Forest plots depicting the effect of day of post-injury strength assessment on effect size: **a** studies using human subjects; **b** studies using animal subjects; **c** studies with data for all 3 days. The *center of a diamond* represents the subgroup effect size for a given day. *Diamond width* represents the 95 % confidence interval for the subgroup effect size. The number of independent groups contributing to a subgroup effect size is listed within the *parentheses*. An *asterisk* indicates a significant difference between subgroups analyzed using post hoc pairwise comparisons and a Benjamini and Hochberg false discovery rate-adjusted α level. *CI* confidence interval, *ES* effect size

The day of post-injury strength assessment was a significant moderator variable. In the analysis examining all studies (Table 1) and in the analysis examining human studies only (Fig. 3a), studies making measurements of strength at day 3 post-injury had an ES that was greater than that for studies taking measurements at day 2 post-injury, which in turn was greater than that for studies taking measurements at day 1 post-injury. The moderator variable’s effect was different when analyzing animal studies only (Fig. 3b). Only animal studies taking measurements at day 3 post-injury had an ES that was significantly less than the ES for studies taking measurements at day 2 post-injury (+0.44 vs. +1.19; *P* = 0.02). More importantly, the ES for each of the day subgroups were positive and statistically greater than zero. When the analysis was run using only studies, including human and animal studies, that took measurements on all 3 days, the findings were identical to those for all studies and the human-only studies (Fig. 3c).

To determine if the muscle group injured could explain some of the between-study variation in ES, subgroup meta-analyses were performed separately for human and animal studies (Table 1). For humans, the ESs from studies injuring elbow flexors, knee extensors, and/or knee flexors were compared. For animals, ankle plantarflexor muscles were contrasted against ankle dorsiflexor muscles. The muscle group tested was a significant moderator variable for both the human and animal studies. The ES for the group of studies injuring the elbow flexors was three to four times greater than the ES for the groups of studies injuring the knee extensors or flexors. These data suggest that the strength recovery over the first 3 days post-injury may be greater for the elbow flexors than for either the knee extensors or flexors. In the animal studies using the ankle plantarflexors, the ES was approximately three times greater than the ES for the studies using the dorsiflexors. This suggests that the plantarflexors may recover strength faster following injury.

The injury induced in all human studies was induced using eccentric contractions or eccentric contraction-biased exercise but several injury models were employed in the animal studies including eccentric contraction-induced injury, freeze injury, crush injury, and ischemia–reperfusion injury. To determine if the type of injury could explain some of the between-study variation in the ES, a subgroup meta-analysis was performed for animal studies comparing eccentric contraction-induced injury models with traumatic injury models (i.e., freeze, crush, and ischemia–reperfusion models combined). However, injury type was not found to be a significant moderator variable (*P* = 0.78) (Table 1). In addition, the type of contraction used to assess strength (i.e., isometric vs. isokinetic or isotonic) was unable to explain any heterogeneity (*P* = 0.67).

The presence of fatigue in a study at the time of the immediate post-injury assessment was able to explain a significant amount of the between-study variation in ES (*P* = 0.03) (Table 1). When fatigue was present, the study ES was greater than when the fatigue state in a study was unknown (+0.62 vs. +0.30). Similarly, when fatigue was present, the study ES approached being significantly greater than the ES from studies where fatigue was not present or unlikely to be present (+0.62 vs. +0.32; *P* = 0.054).

**Fig. 4:**
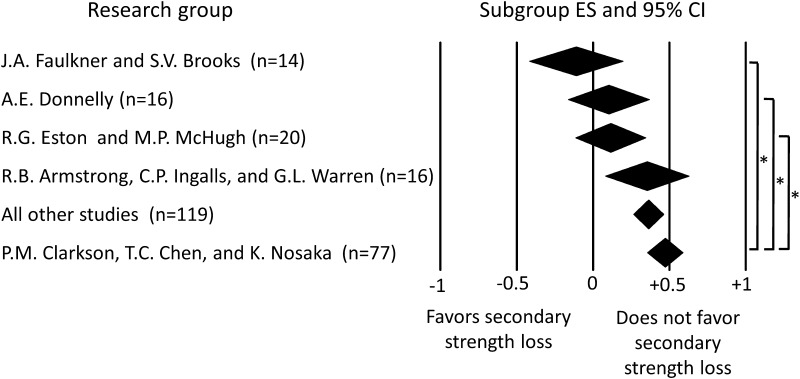
Forest plot depicting the effect of research group on effect size. The *center of a diamond* represents the effect size for a given research group. *Diamond width* represents the 95 % confidence interval for the subgroup effect size. Research group assignments were made after careful cross-of all studies to detect collaborations (e.g., co-authorships) and shared research approaches (e.g., same or similar experimental model). For consideration as a group for the subgroup meta-analysis, each research group had to have a minimum of ten independent groups of subjects included in the overall meta-analysis. Studies not assigned to a research group were lumped together in the “All other studies” group. The number of independent groups contributing to a subgroup effect size is listed within the *parentheses*. An *asterisk* indicates a significant difference between subgroups analyzed using post hoc pairwise comparisons and a Benjamini and Hochberg false discovery rate-adjusted α level. *CI* confidence interval, *ES* effect size

A subgroup meta-analysis conducted to determine if the research group performing a study could explain some of the between-study variation in ES yielded statistically significant results (*P* = 0.001) (Fig. 4). Research group ES values ranged from −0.11 for the “J. A. Faulkner and S. V. Brooks” group to +0.47 for the “P.M. Clarkson, T. C. Chen, and K. Nosaka” group. The “J. A. Faulkner and S. V. Brooks” group was the only research group to have a negative ES, though it was not statistically less than zero. Post hoc testing revealed that there were three pairs of groups, with one group being significantly different from the other (Fig. 4).

Meta-regression analysis was used to determine if two continuous variables, study publication year and subject age, could explain any of the between-study variation in ES. There was no significant (*P* = 0.17) linear relationship between the year a study was published and its ES (slope = +0.008 year^−1^; 95 % CI −0.004 to 0.021). The analysis of subject age was run separately for human and animal studies. There was no significant (*P* ≥ 0.16) linear relationship between mean subject age in years and study ES when analyzing either human studies (slope = +0.006 year^−1^; 95 % CI −0.005 to 0.017) or animal studies (slope = −0.319 year^−1^; 95 % CI −0.766 to 0.129).

**Fig. 5:**
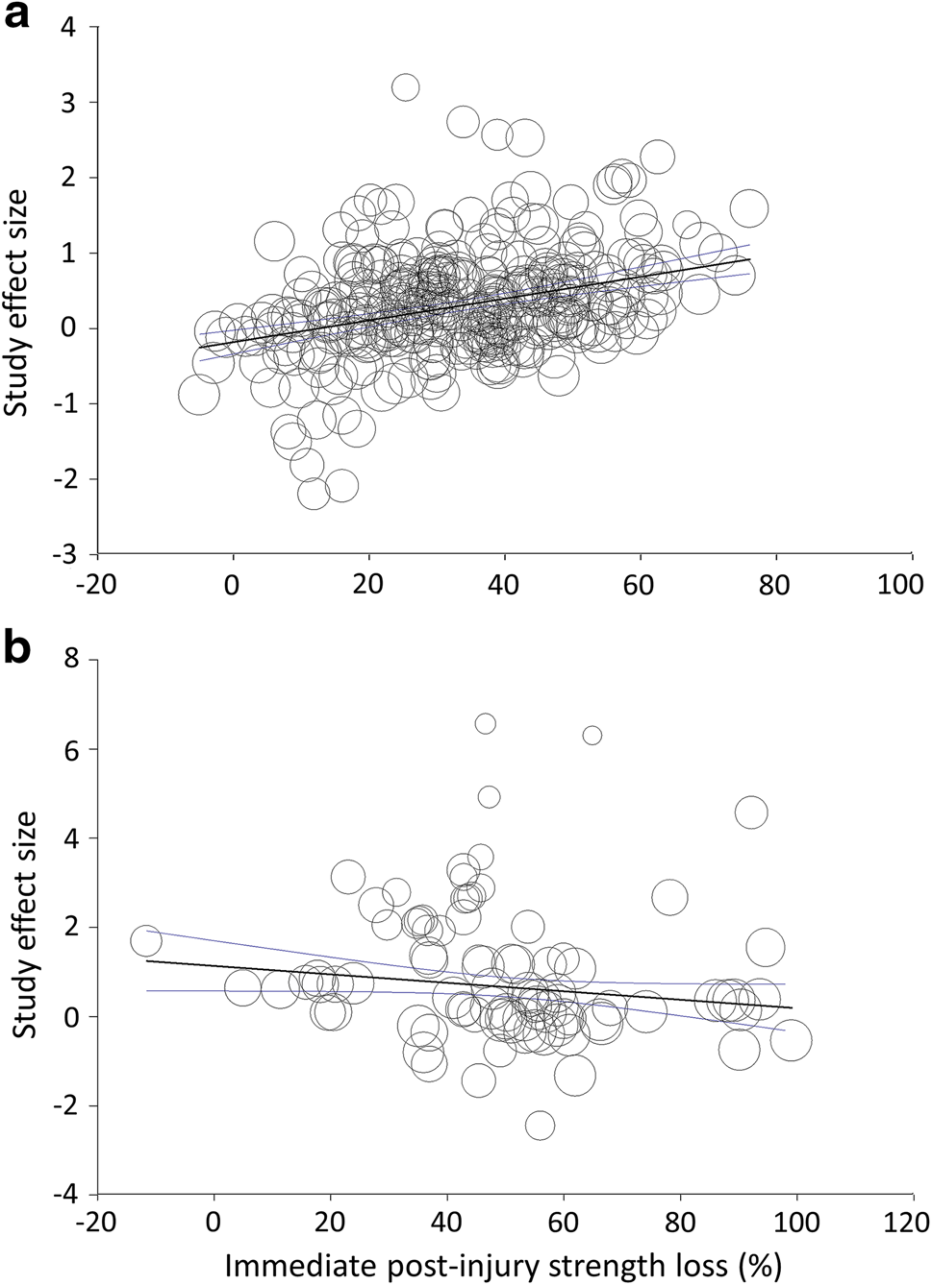
Meta-regression analysis of the relationship between the magnitude of the immediate post-injury strength loss (%) and study (or independent group) effect size for human-only studies (**a**) and animal-only studies (**b**). Each study or independent group is represented by a *circle* and the *size of a circle* reflects the degree of weighting for that datapoint. There are 334 and 88 datapoints in (**a**) and (**b**), respectively. These outnumber the numbers of studies and independent groups because many studies measured strength in more than one fashion. In (**a**) and (**b**), the *straight line* reflects the line of best fit and is surrounded by two *curvilinear lines* representing the 95 % confidence interval. The statistical significance of the relationships in (**a**) and (**b**) are *P* < 0.000000001 and *P* = 0.02, respectively

Meta-regression was also used to determine the relationship between the mean immediate post-injury strength loss (%) and study ES. One might hypothesize that a secondary strength loss would be more likely to occur with a greater initial injury. Figure 5 illustrates the relationship for both the studies using humans (Fig. 5a) and those using animals (Fig. 5b). Surprisingly, the relationship was positive in the human studies (*P* < 0.000000001) but negative in the animal studies (*P* = 0.02). More importantly, predicted study ES was only negative when the immediate post-injury strength loss was small (i.e., <13 %) in the human studies. In contrast, the predicted study ES in the animal studies was never less than zero. The smallest predicted ES would be +0.19, occurring at a 100 % immediate post-injury strength loss. If the studies exhibiting fatigue at the time of the immediate post-injury measurement were removed from the analyses, the relationship between the immediate post-injury strength loss and study ES was lost in the animal studies but remained unchanged in the human studies.

## Discussion

Overall, we found 262 independent groups of subjects from 223 studies in which strength was measured immediately post-injury and again at 1, 2, and/or 3 days post-injury. Our analysis was based on a total of 936 ESs, which were calculated from a total of over 4000 human and animal subjects. The calculation of the ES was designed so that a negative ES would be interpreted as supporting a secondary strength loss. Furthermore, in order to establish a significant secondary strength loss, a negative ES had to be statistically less than zero. Our meta-analysis of the data from the 223 studies yielded a small-to-medium, positive overall ES that was statistically greater than zero (*P* < 0.00000001) (Electronic Supplementary Material Figure S1), indicating that strength in the typical study recovers during the first 3 days after injury. However, there was a large variation in ES among studies (i.e., *I* ^2^ = 86 %). Experimental factors that could explain the variability, at least partially, included the sex of the subject, the day of post-injury strength assessment, whether fatigue was present immediately post-injury, the muscle group injured, the research group conducting the study, and the magnitude of the immediate post-injury strength loss (Table 1; Figs. 3, 4, 5).

### Technical Limitations of the Analysis

Potential technical limitations of our systematic review and meta-analysis include (1) the likelihood of our review having missed studies; and (2) failure to know the between-trial correlations in many studies utilizing a repeated-measures research design. We performed an extensive literature search for studies conducted in 2011 or before. We did not exclude unpublished studies, non-English studies, or studies based on geographical location. However, it is doubtful that our systematic review retrieved all relevant studies and therefore our analysis probably consists of a random subset of all relevant studies. While failure to include all possible studies can affect meta-analysis statistical power and ES CIs, we do not believe that this limitation had a significant impact on the overall ES [12]. An Orwin’s Fail-Safe N test was used to determine how many missing studies would have to exist in order to bring the overall ES down to a level indicating a secondary strength loss [16]. We sought to identify the number of missing studies that would need to be *found* to produce a negative, albeit trivial, overall effect (i.e., ES = −0.1) that would be statistically less than zero. Assuming that the missing studies had a mean ES of −0.5, we determined that 234 missing studies would have to be found and added to our meta-analysis before we could conclude that, on average, a secondary strength loss occurs following injury. This number of missing studies is almost equal to the total number of independent groups that we did find. Furthermore, only 8 % of the 262 independent groups we retrieved had an ES of −0.5 or less. We believe it is highly unlikely that we missed 234 or more studies with a mean ES of –0.5 and thus this analysis is consistent with the argument that a secondary strength loss does not occur in the typical study.

The other technical limitation of our systematic review and meta-analysis was not knowing the between-trial correlations (i.e., that between immediate post-injury and the day 1–3 post-injury measures) in most of the studies employing a repeated-measures research design. We were only able to calculate correlations for 14 independent groups and used the median of those 14 correlations (i.e., 0.803) as the correlation for the groups for which a correlation could not be calculated. The inability to calculate between-trial correlations is a common issue for data extraction from primary research studies but the effect of this limitation can be addressed by performing a sensitivity analysis [12]. We performed a sensitivity analysis by allowing the assumed correlation to vary between what we considered to be the extreme possibilities for the correlation. By varying the correlation between 0 and 0.95, the overall ES varied minimally from +0.299 to +0.339 while remaining statistically greater than zero (*P* < 0.00000001) in all instances. Therefore, our sensitivity analysis indicates that not knowing the between-trial correlations for all studies most likely had minimal effect on the overall ES and its qualitative label as a small-to-medium, positive effect.

### Evidence For and Against Secondary Strength Loss

There was minimal evidence in our analysis supporting a secondary strength loss after muscle injury, even when considering the potential moderator variables that might explain some of the marked between-study variation in ES. For example, the subgroup meta-analysis probing how the day of post-injury assessment affected study ESs showed that for both all studies and the human-only studies, the ES for the subgroup of studies measuring strength at 3 days post-injury was significantly larger than that for the subgroup measuring strength at 2 days post-injury, which in turn was larger than that for the subgroup measuring strength at 1 day post-injury (Table 1; Fig. 3a). These results were the same as when we conducted a subgroup meta-analysis using only studies that made measurements on all 3 days (Fig. 3c). While causal conclusions cannot be made from subgroup meta-analyses, this particular analysis presents the strongest case for the interpretation that muscle strength is steadily recovering over the first 3 days post-injury.

Overall, we looked at ten moderator variables using the subgroup meta-analysis procedure. This generated a total of 36 subgroups and there was only one subgroup with a negative ES, and that ES was not statistically less than zero (Table 1; Figs. 3, 4). Moreover, of the 35 subgroups with positive ESs, all but six were statistically greater than zero. From the 223 studies included in our review, we also conducted an overall meta-analysis using the smallest (or most negative) ES from each study and still observed a positive overall effect (ES = +0.067), which was statistically greater than zero. Given the criterion to establish a secondary strength loss (i.e., a negative ES that was statistically less than zero), this “smallest ES” analysis approach was biased towards finding a secondary strength loss but still failed to do so. Also, the mean immediate post-injury strength loss among the 223 studies varied substantially, and one might posit that greater initial injury would coincide with the appearance of a secondary strength loss. However, the relationships between mean immediate post-injury strength loss and study ESs determined by meta-regressions were not predictive of a secondary strength loss in the case of the animal-only studies or were only predictive of a secondary strength loss at relatively small immediate post-injury strength losses in the human-only studies (Fig. 5). Collectively, we found minimal evidence to support a statistically significant secondary strength loss.

### Explanations for Between-Study Variance in Effect Size

Over the past 30 years, a number of research groups have made significant contributions to the understanding of muscle injury and repair. It is not surprising that differences in ES were observed among the five research groups in our “research group” analysis because these groups have used different experimental models and had differing research objectives. For example, of the five groups, the “J. A. Faulkner and S. V. Brooks” and “R. B. Armstrong, C. P. Ingalls, and G. L. Warren” groups primarily used rodent models, with the former group utilizing an in situ model for inducing injury versus an in vivo model being used by the latter group. The three other research groups primarily used human subjects, with the “P. M. Clarkson, T. C. Chen, and K. Nosaka” studies typically injuring the elbow flexors while the “A. E. Donnelly” and “R. G. Eston and M. P. McHugh” studies typically injured the knee extensors and/or flexors. It is important to remind the reader that in a separate subgroup meta-analysis we found human studies injuring the elbow flexors to have an ES that was much greater than that for studies injuring the knee extensors. However, it is not possible to determine if this *muscle group* difference is truly a muscle group difference or if it occurred due to other experimental differences among the “P. M. Clarkson, T. C. Chen, and K. Nosaka,” “A. E. Donnelly,” and “R. G. Eston and M. P. McHugh” research groups. This emphasizes why causal conclusions cannot be made from the results of subgroup meta-analyses or meta-regressions. However, what is clear from this “research group” analysis is that no single subgroup ES was statistically less than zero, which argues against a secondary loss of strength occurring.

In a narrative review discussing secondary strength loss, Faulkner and associates [3] wrote that the magnitude of the primary injury can be determined by measuring strength loss at 3 h post-injury to rule out any contribution to strength loss arising from fatigue. While both injury and fatigue can cause an immediate loss of muscle strength, fatigue is characterized by the reversal of strength loss with rest, whereas muscle injury requires muscle fiber repair and/or regeneration and strength recovery is more prolonged. Per our inclusion criteria, we accepted studies with strength measurements up to 6 h post-injury for our “immediate post-injury” timepoint. We subsequently performed a subgroup meta-analysis to determine the potential impact that fatigue could have had on the study ES. Specifically, if fatigue contributed to the immediate post-injury strength loss, this would make it more difficult to detect a secondary strength loss because the ES would be inflated. Indeed, the ES for studies where fatigue was present was twofold greater than the ES for all other studies (i.e., +0.62 vs. +0.30–0.32). More importantly, this subgroup meta-analysis also showed that when fatigue was not present or not likely to be present, there was still a positive ES that was statistically greater than zero (Table 1). We concluded that fatigue can confound the strength loss immediately post-injury in eccentric contraction-induced injury studies and thus inflate the associated ES. However, fatigue appears to have occurred in a minority of studies and thus it is unlikely to have substantially affected our overall finding and interpretation of that finding.

The results of our “day of post-injury strength assessment” subgroup meta-analysis conducted on animal-only studies (Fig. 3b) may lead some to suggest that we should give consideration to calculating the ES for strength data collected after 3 days post-injury. This is because the studies taking measurements of strength at 3 days post-injury had a significantly lower ES than that of studies taking measurements at 2 days. One might hypothesize that the ES could decline further over the next few days post-injury, eventually becoming negative. However, using data collected after 3 days post-injury is outside the time frame originally proposed for when a secondary strength loss occurs [3]. Furthermore, as compared with the analysis using human-only studies, there were far fewer independent groups contributing to the animal-only subgroup meta-analysis (i.e., 11–40 vs. 163–192 studies per timepoint). With far fewer studies in the animal-only analysis, it is more likely for a subgroup ES to be influenced by a particular research group’s approach or methodology. For example, 15 of the 40 “3 days post-injury” ESs came from the “J. A. Faulkner and S. V. Brooks” research group, whereas only one of the 11 “2 days post-injury” ESs came from that group. This is noteworthy because the “J. A. Faulkner and S. V. Brooks” studies tended to have negative ESs (Fig. 4), and thus this might help explain why the “3 days post-injury” ES was lower than that for 2 days post-injury.

The present analysis provides insight for future research directions. In particular, studies exploring subject sex differences or muscle group differences in the strength recovery from muscle injury would be warranted (Table 1). One interpretation from the “subject sex” subgroup meta-analysis is that males recover faster than females. Alternatively, males could be more susceptible to muscle injury (i.e., greater immediate strength loss) and if males and females recovered to a similar percentage of initial strength by 3 days post-injury, the rate of strength recovery would appear to be greater for males. This theory would tend to agree with our meta-regression analysis showing a positive relationship between immediate strength loss and the study ES in the human-only studies (Fig. 5a). However, assumptions on sex differences in susceptibility or recovery from injury are not supported by a cursory review of the literature, at least for human studies [17]. Similar to subject sex differences in ES, the greater ES for human studies employing the elbow flexors may simply reflect how the ES is calculated because the elbow flexors appear to be more susceptible to eccentric contraction-induced injury than are the knee extensors [18–20]; the immediate strength loss for the elbow flexors is ~50 % greater on average [18]. A possible alternative explanation for the muscle group difference is that the elbow flexors may be more fatigable than the knee extensors during the performance of eccentric contractions. However, in our analysis of the incidence of fatigue, the association of fatigue with studies using the elbow flexors was no different from that for studies using the knee extensors (*P* = 0.92).

### Practical and Clinical Implications

A primary motivation for this meta-analysis was to determine if a secondary loss of strength occurs after the initiating injurious event so that one can make more informed decisions about if and how to treat muscle injuries. Specifically, the autogenetic and inflammatory phases following injury have been directly implicated as causing a secondary deleterious event in skeletal muscle leading to a secondary loss of strength [3, 14, 21, 22]. If such a secondary strength loss occurs, then pharmacological, dietary, and therapeutic interventions to block autogenetic pathways and/or inflammation in the first few days post-injury might be efficacious. On the other hand, if there is no secondary strength loss, more caution should be employed for such interventions because they might impair the recovery process. The results of our systematic review and meta-analysis may suggest why pharmacological, dietary, and therapeutic interventions have not been generally effective [23, 24], i.e., because there is minimal, if any, evidence for a secondary injury, at least not an injury resulting in an additional strength loss. This could be because the autogenetic and inflammatory phases may be constrained to lie within the initially damaged tissue and thus may be thought of as parts of the tissue repair and regeneration processes rather than degeneration processes that cause additional damage.

We can also envision a scenario in which secondary injury occurs but a secondary strength loss does not. We have shown that in the first few days after the initiation of eccentric contraction-induced injury, most (i.e., 50–75 %) of the strength loss is attributable to a failure in the excitation–contraction (E-C) coupling process, with the remainder of the strength loss being due to physical disruption of force-bearing elements within the muscle [25]. The strength loss attributable to E-C uncoupling is recovered more rapidly than that associated with physical disruption. We have hypothesized that the former recovery process is more of a repair process and does not require degeneration and subsequent regeneration of muscle fibers, whereas recovery of strength from physical disruption would require degeneration, including inflammation, and regeneration [26]. We theorize that it is possible to have some damaged muscle fibers recovering from E-C uncoupling at the same time that other damaged fibers, although fewer in number, are engulfed in inflammatory infiltrate to the extent that adjacent, but previously undamaged, fibers incur a bystander injury. In this scenario, strength measured at the whole muscle level could be recovering but locally, initially uninjured fibers could be undergoing damage by the inflammation but not to the extent that the muscle strength recovery is blunted or noticeably slowed. Support for such a hypothesis comes from a study by Pizza and colleagues [27] in which eccentric contraction-induced injury was induced in muscles of wild-type mice and mice with a genetic deletion that blunts neutrophil accumulation in injured muscle. There was no evidence of a secondary strength loss in either strain of mice, i.e., strength recovered steadily over time after injury, but strength recovered faster in the mice with a reduced neutrophil accumulation in the injured muscle. The scenario of having a secondary injury without a secondary strength loss can easily be envisioned when the mechanisms for injury, and the recovery from it, are not homogenous, either temporally or spatially, within the muscle.

## Conclusions

A robust systematic review and meta-analysis was conducted utilizing data from 262 independent groups of subjects reported in 223 studies with over 4000 subjects and 936 separate ESs. Our findings do not support the presence of a secondary strength loss following an initial injurious event in skeletal muscle. In fact, our findings suggest that, on average, strength recovers steadily over the first 3 days post-injury, particularly in humans. Moving forward, we recommend that future studies no longer use secondary strength loss as a foundation on which to justify a study. Furthermore, careful thought should be applied before initiating a study using an intervention (e.g., dietary, pharmacological, therapeutic) to block or attenuate inflammatory or autogenetic processes when recovery of muscle strength is desired.

## Electronic supplementary material

Below is the link to the electronic supplementary material.

Supplementary material 1 (PDF 390 kb)
