# Supplementary material for: Minimal Evidence for a Secondary Loss of Strength After an Acute Muscle Injury: A Systematic Review and Meta-Analysis
Source: Sports Med. 2016 Apr 21;47(1):41–59. doi: 10.1007/s40279-016-0528-7 (PMC5214801; doi:10.1007/s40279-016-0528-7)
Supplement: Supplementary file 1 — Supplementary material 1 (PDF 390 kb) [file 40279_2016_528_MOESM1_ESM.pdf]

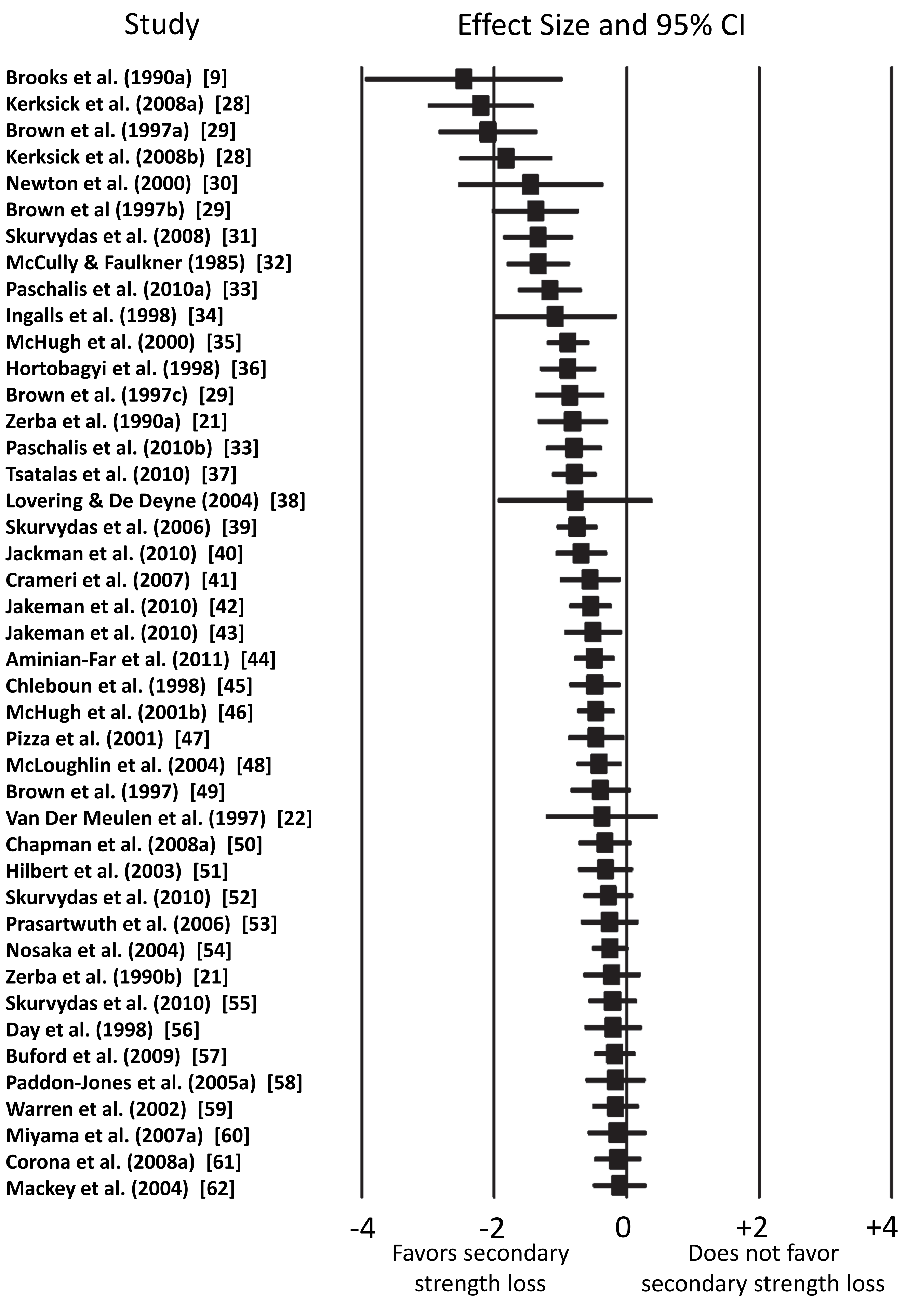
Study
Effect Size and 95% CI
Brooks et al. (1990a) [9]
Kerksick et al. (2008a) [28]
Brown et al. (1997a) [29]
Kerksick et al. (2008b) [28]
Newton et al. (2000) [30]
Brown et al (1997b) [29]
Skurvydas et al. (2008) [31]
McCully & Faulkner (1985) [32]
Paschalis et al. (2010a) [33]
Ingalls et al. (1998) [34]
McHugh et al. (2000) [35]
Hortobagyi et al. (1998) [36]
Brown et al. (1997c) [29]
Zerba et al. (1990a) [21]
Paschalis et al. (2010b) [33]
Tsatalas et al. (2010) [37]
Lovering & De Deyne (2004) [38]
Skurvydas et al. (2006) [39]
Jackman et al. (2010) [40]
Crameri et al. (2007) [41]
Jakeman et al. (2010) [42]
Jakeman et al. (2010) [43]
Aminian-Far et al. (2011) [44]
Chleboun et al. (1998) [45]
McHugh et al. (2001b) [46]
Pizza et al. (2001) [47]
McLoughlin et al. (2004) [48]
Brown et al. (1997) [49]
Van Der Meulen et al. (1997) [22]
Chapman et al. (2008a) [50]
Hilbert et al. (2003) [51]
Skurvydas et al. (2010) [52]
Prasartwuth et al. (2006) [53]
Nosaka et al. (2004) [54]
Zerba et al. (1990b) [21]
Skurvydas et al. (2010) [55]
Day et al. (1998) [56]
Buford et al. (2009) [57]
Paddon-Jones et al. (2005a) [58]
Warren et al. (2002) [59]
Miyama et al. (2007a) [60]
Corona et al. (2008a) [61]
Mackey et al. (2004) [62]
-4
-2
0
+2
+4
Favors secondary strength loss
Does not favor secondary strength loss

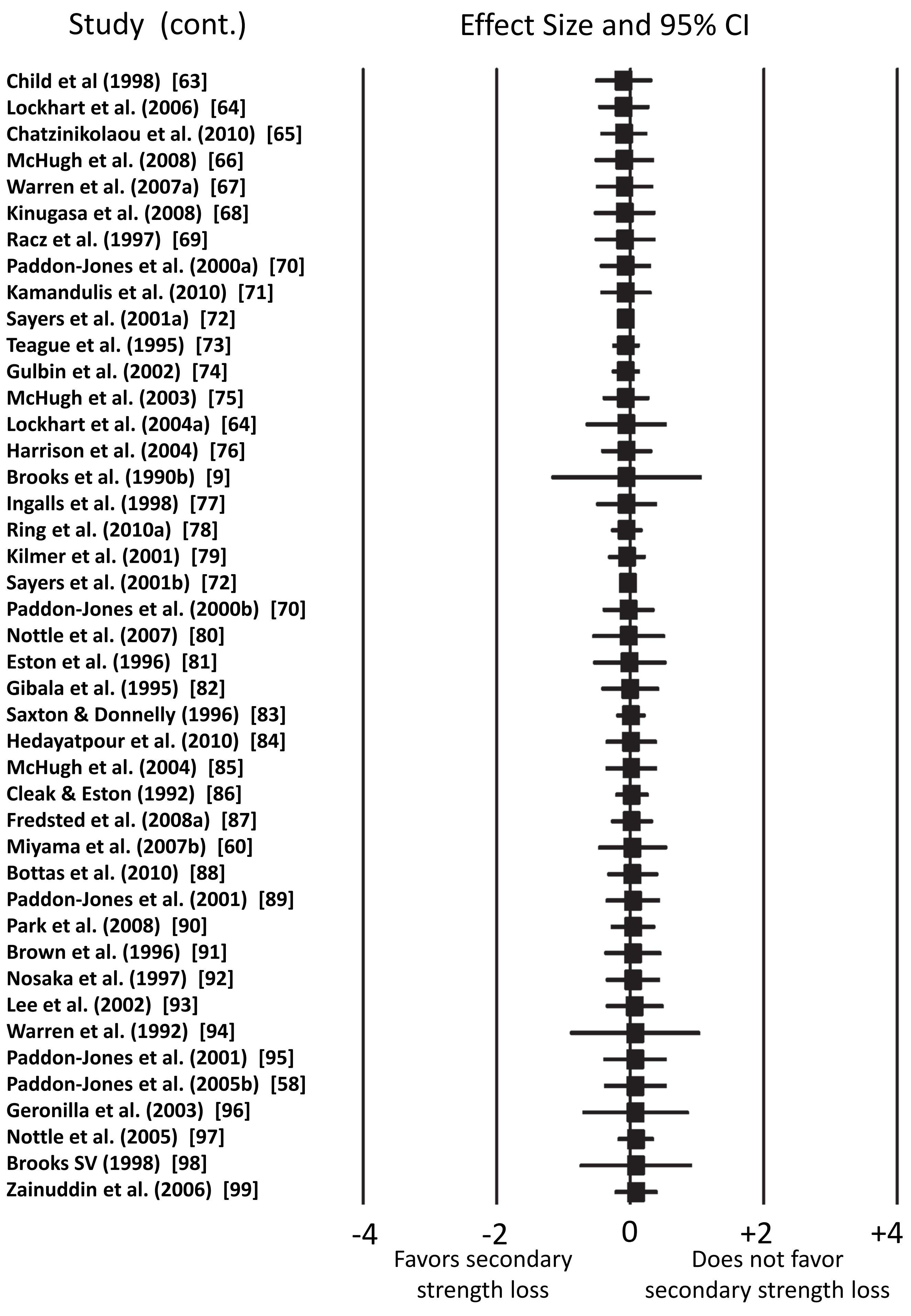
Study (cont.)
Effect Size and 95% CI
Child et al (1998) [63]
Lockhart et al. (2006) [64]
Chatzinikolaou et al. (2010) [65]
McHugh et al. (2008) [66]
Warren et al. (2007a) [67]
Kinugasa et al. (2008) [68]
Racz et al. (1997) [69]
Paddon-Jones et al. (2000a) [70]
Kamandulis et al. (2010) [71]
Sayers et al. (2001a) [72]
Teague et al. (1995) [73]
Gulbin et al. (2002) [74]
McHugh et al. (2003) [75]
Lockhart et al. (2004a) [64]
Harrison et al. (2004) [76]
Brooks et al. (1990b) [9]
Ingalls et al. (1998) [77]
Ring et al. (2010a) [78]
Kilmer et al. (2001) [79]
Sayers et al. (2001b) [72]
Paddon-Jones et al. (2000b) [70]
Nottle et al. (2007) [80]
Eston et al. (1996) [81]
Gibala et al. (1995) [82]
Saxton & Donnelly (1996) [83]
Hedayatpour et al. (2010) [84]
McHugh et al. (2004) [85]
Cleak & Eston (1992) [86]
Fredsted et al. (2008a) [87]
Miyama et al. (2007b) [60]
Bottas et al. (2010) [88]
Paddon-Jones et al. (2001) [89]
Park et al. (2008) [90]
Brown et al. (1996) [91]
Nosaka et al. (1997) [92]
Lee et al. (2002) [93]
Warren et al. (1992) [94]
Paddon-Jones et al. (2001) [95]
Paddon-Jones et al. (2005b) [58]
Geronilla et al. (2003) [96]
Nottle et al. (2005) [97]
Brooks SV (1998) [98]
Zainuddin et al. (2006) [99]
-4
-2
0
+2
+4
Favors secondary strength loss
Does not favor secondary strength loss

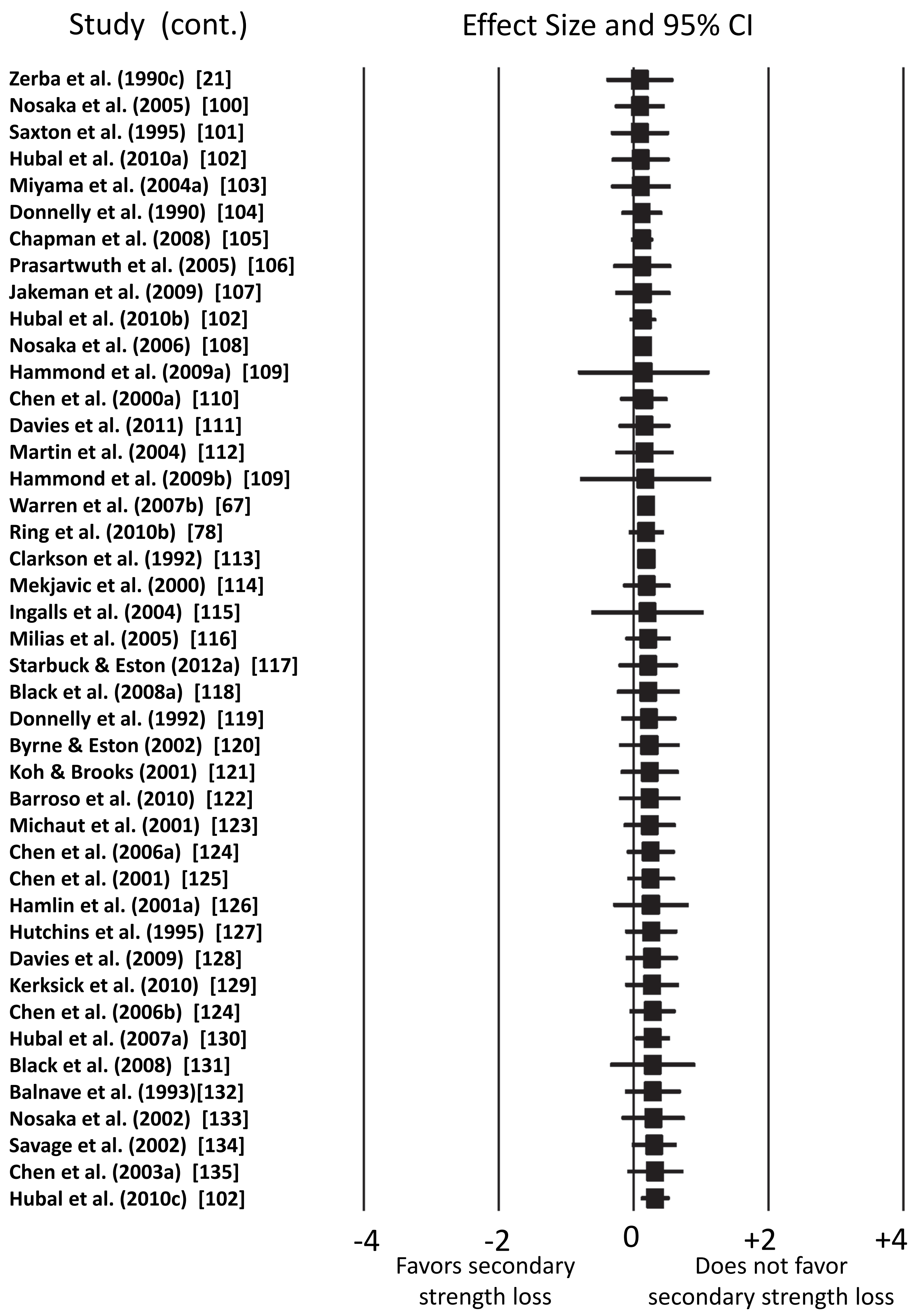
Study (cont.)
Effect Size and 95% CI
Zerba et al. (1990c) [21]
Nosaka et al. (2005) [100]
Saxton et al. (1995) [101]
Hubal et al. (2010a) [102]
Miyama et al. (2004a) [103]
Donnelly et al. (1990) [104]
Chapman et al. (2008) [105]
Prasartwuth et al. (2005) [106]
Jakeman et al. (2009) [107]
Hubal et al. (2010b) [102]
Nosaka et al. (2006) [108]
Hammond et al. (2009a) [109]
Chen et al. (2000a) [110]
Davies et al. (2011) [111]
Martin et al. (2004) [112]
Hammond et al. (2009b) [109]
Warren et al. (2007b) [67]
Ring et al. (2010b) [78]
Clarkson et al. (1992) [113]
Mekjavic et al. (2000) [114]
Ingalls et al. (2004) [115]
Milias et al. (2005) [116]
Starbuck & Eston (2012a) [117]
Black et al. (2008a) [118]
Donnelly et al. (1992) [119]
Byrne & Eston (2002) [120]
Koh & Brooks (2001) [121]
Barroso et al. (2010) [122]
Michaut et al. (2001) [123]
Chen et al. (2006a) [124]
Chen et al. (2001) [125]
Hamlin et al. (2001a) [126]
Hutchins et al. (1995) [127]
Davies et al. (2009) [128]
Kerksick et al. (2010) [129]
Chen et al. (2006b) [124]
Hubal et al. (2007a) [130]
Black et al. (2008) [131]
Balnave et al. (1993)[132]
Nosaka et al. (2002) [133]
Savage et al. (2002) [134]
Chen et al. (2003a) [135]
Hubal et al. (2010c) [102]
-4
-2
0
+2
+4
Favors secondary strength loss
Does not favor secondary strength loss

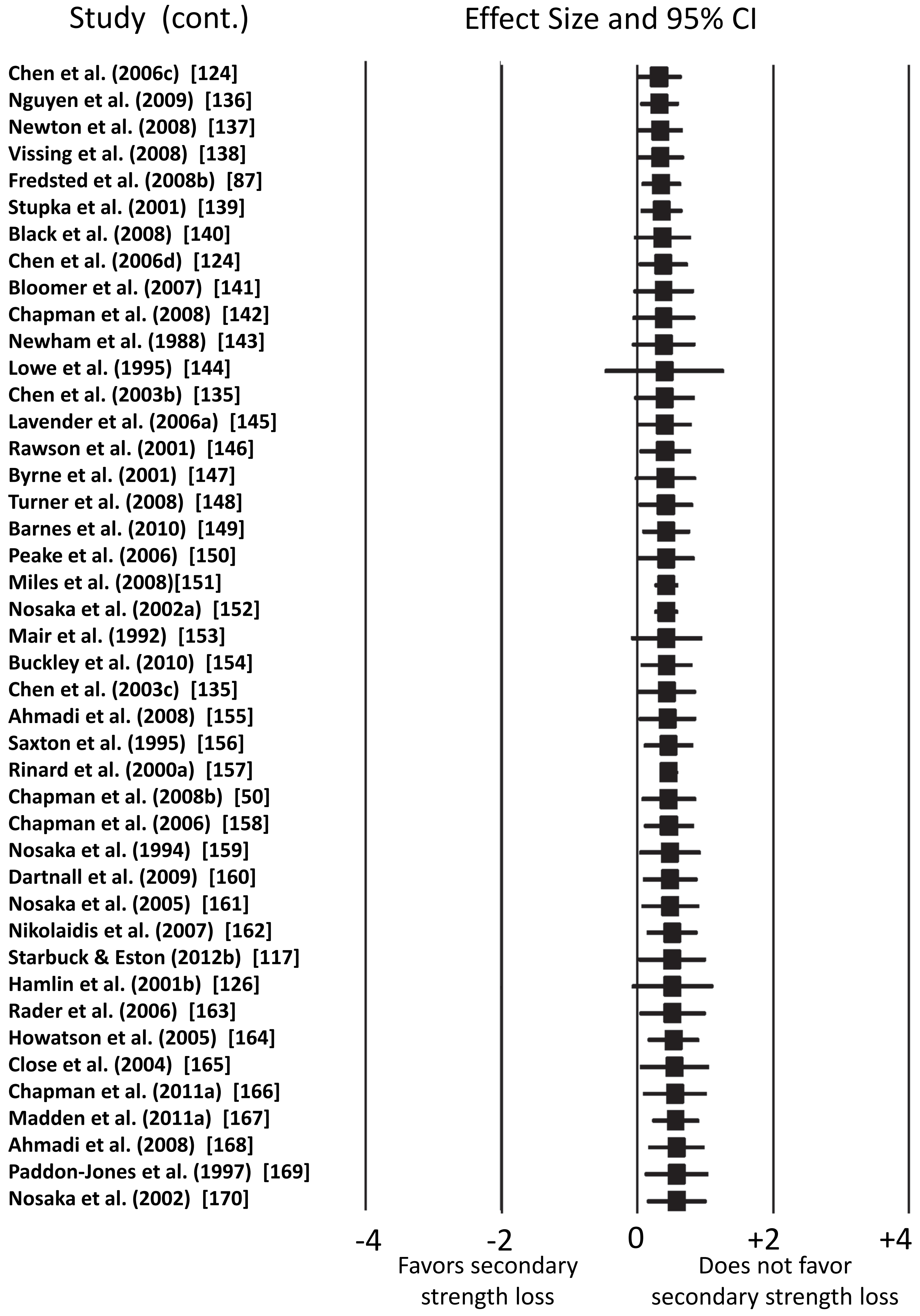
Study (cont.)
Effect Size and 95% CI
Chen et al. (2006c) [124]
Nguyen et al. (2009) [136]
Newton et al. (2008) [137]
Vissing et al. (2008) [138]
Fredsted et al. (2008b) [87]
Stupka et al. (2001) [139]
Black et al. (2008) [140]
Chen et al. (2006d) [124]
Bloomer et al. (2007) [141]
Chapman et al. (2008) [142]
Newham et al. (1988) [143]
Lowe et al. (1995) [144]
Chen et al. (2003b) [135]
Lavender et al. (2006a) [145]
Rawson et al. (2001) [146]
Byrne et al. (2001) [147]
Turner et al. (2008) [148]
Barnes et al. (2010) [149]
Peake et al. (2006) [150]
Miles et al. (2008)[151]
Nosaka et al. (2002a) [152]
Mair et al. (1992) [153]
Buckley et al. (2010) [154]
Chen et al. (2003c) [135]
Ahmadi et al. (2008) [155]
Saxton et al. (1995) [156]
Rinard et al. (2000a) [157]
Chapman et al. (2008b) [50]
Chapman et al. (2006) [158]
Nosaka et al. (1994) [159]
Dartnall et al. (2009) [160]
Nosaka et al. (2005) [161]
Nikolaidis et al. (2007) [162]
Starbuck & Eston (2012b) [117]
Hamlin et al. (2001b) [126]
Rader et al. (2006) [163]
Howatson et al. (2005) [164]
Close et al. (2004) [165]
Chapman et al. (2011a) [166]
Madden et al. (2011a) [167]
Ahmadi et al. (2008) [168]
Paddon-Jones et al. (1997) [169]
Nosaka et al. (2002) [170]
-4
-2
0
+2
+4
Favors secondary strength loss
Does not favor secondary strength loss

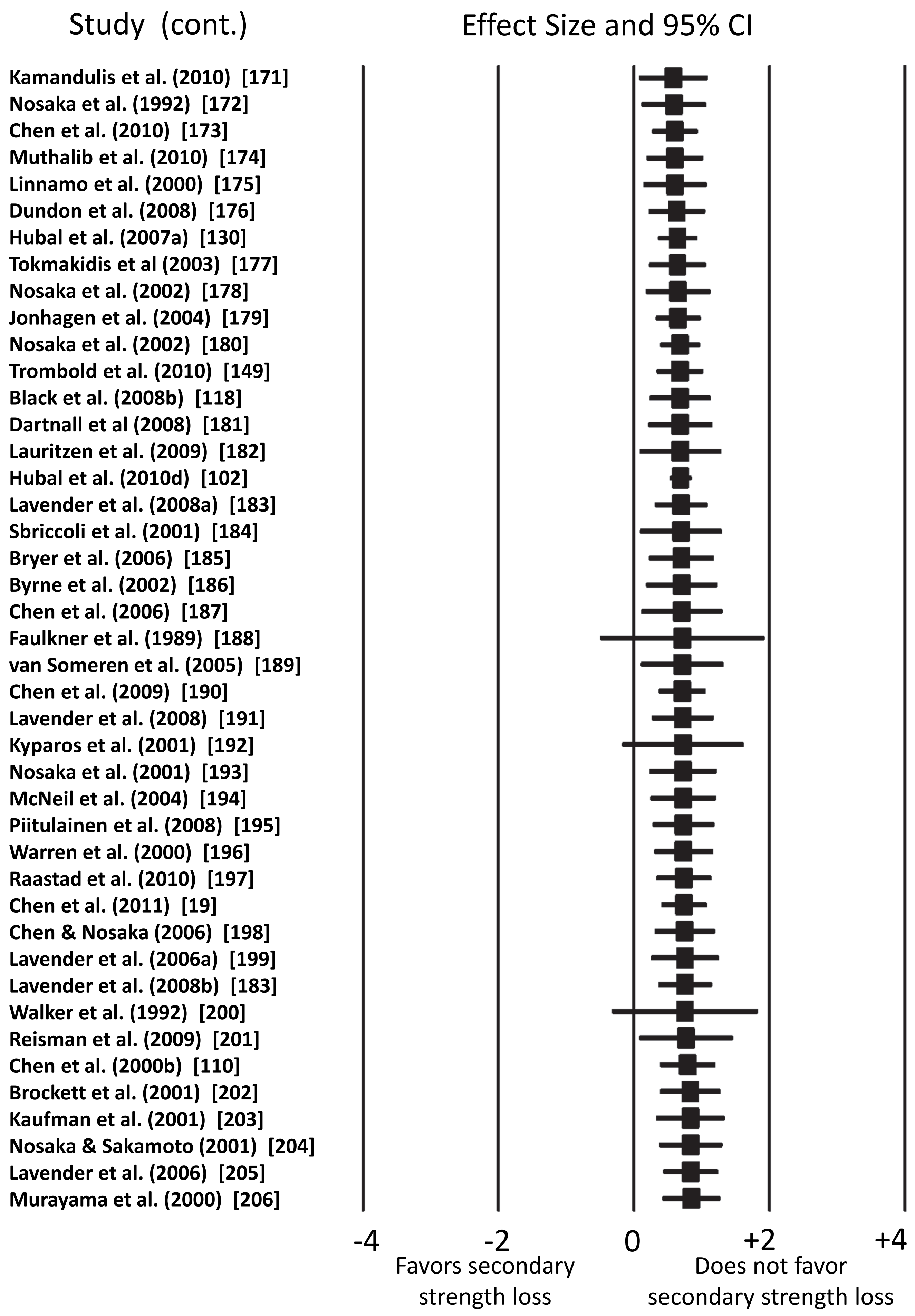
Study (cont.)
Effect Size and 95% CI
Kamandulis et al. (2010) [171]
Nosaka et al. (1992) [172]
Chen et al. (2010) [173]
Muthalib et al. (2010) [174]
Linnamo et al. (2000) [175]
Dundon et al. (2008) [176]
Hubal et al. (2007a) [130]
Tokmakidis et al (2003) [177]
Nosaka et al. (2002) [178]
Jonhagen et al. (2004) [179]
Nosaka et al. (2002) [180]
Trombold et al. (2010) [149]
Black et al. (2008b) [118]
Dartnall et al (2008) [181]
Lauritzen et al. (2009) [182]
Hubal et al. (2010d) [102]
Lavender et al. (2008a) [183]
Sbriccoli et al. (2001) [184]
Bryer et al. (2006) [185]
Byrne et al. (2002) [186]
Chen et al. (2006) [187]
Faulkner et al. (1989) [188]
van Someren et al. (2005) [189]
Chen et al. (2009) [190]
Lavender et al. (2008) [191]
Kyparos et al. (2001) [192]
Nosaka et al. (2001) [193]
McNeil et al. (2004) [194]
Piitulainen et al. (2008) [195]
Warren et al. (2000) [196]
Raastad et al. (2010) [197]
Chen et al. (2011) [19]
Chen & Nosaka (2006) [198]
Lavender et al. (2006a) [199]
Lavender et al. (2008b) [183]
Walker et al. (1992) [200]
Reisman et al. (2009) [201]
Chen et al. (2000b) [110]
Brockett et al. (2001) [202]
Kaufman et al. (2001) [203]
Nosaka & Sakamoto (2001) [204]
Lavender et al. (2006) [205]
Murayama et al. (2000) [206]
-4
-2
0
+2
+4
Favors secondary strength loss
Does not favor secondary strength loss

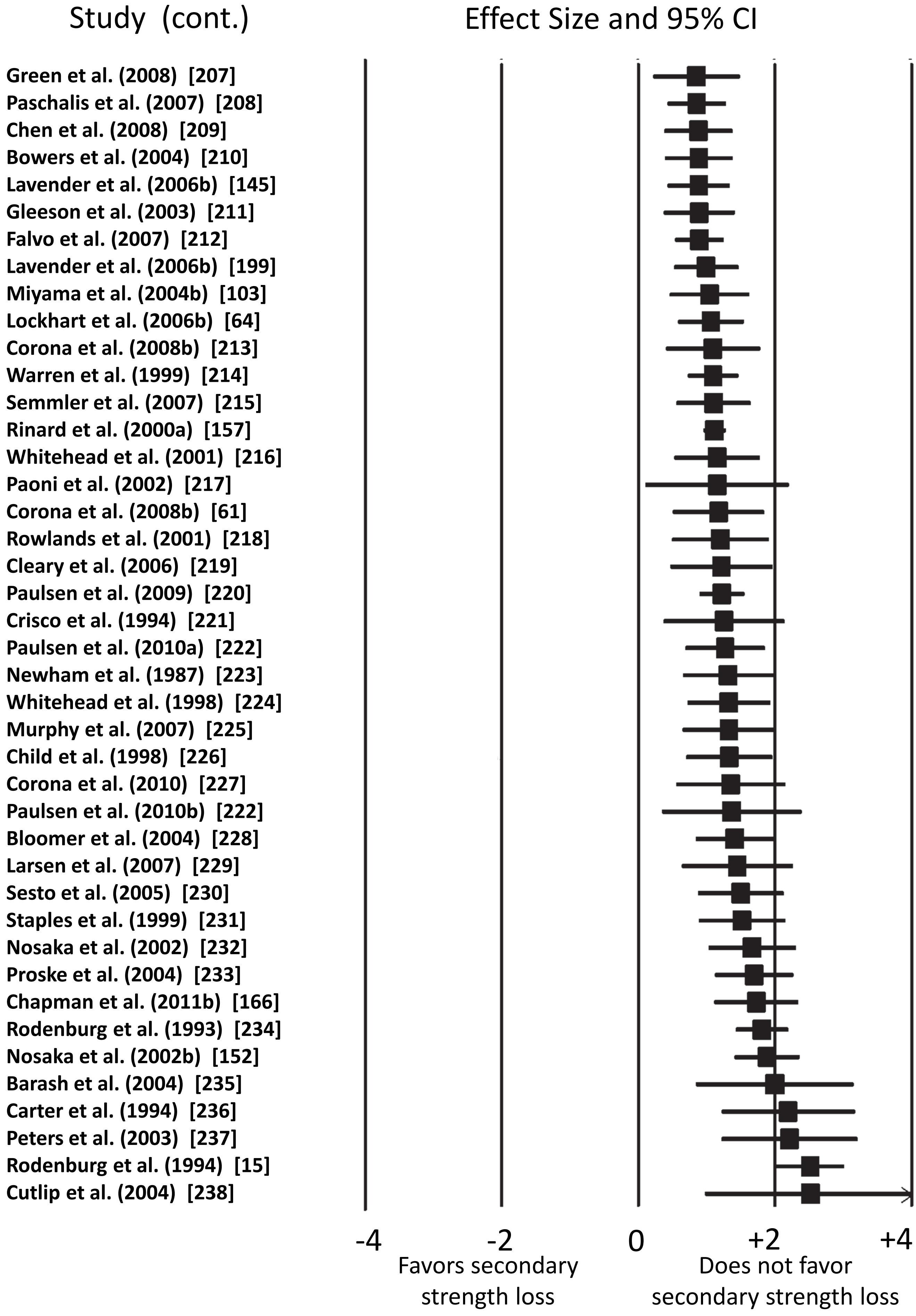

Study (cont.)
Effect Size and 95% CI
Green et al. (2008) [207]
Paschalis et al. (2007) [208]
Chen et al. (2008) [209]
Bowers et al. (2004) [210]
Lavender et al. (2006b) [145]
Gleeson et al. (2003) [211]
Falvo et al. (2007) [212]
Lavender et al. (2006b) [199]
Miyama et al. (2004b) [103]
Lockhart et al. (2006b) [64]
Corona et al. (2008b) [213]
Warren et al. (1999) [214]
Semmler et al. (2007) [215]
Rinard et al. (2000a) [157]
Whitehead et al. (2001) [216]
Paoni et al. (2002) [217]
Corona et al. (2008b) [61]
Rowlands et al. (2001) [218]
Cleary et al. (2006) [219]
Paulsen et al. (2009) [220]
Crisco et al. (1994) [221]
Paulsen et al. (2010a) [222]
Newham et al. (1987) [223]
Whitehead et al. (1998) [224]
Murphy et al. (2007) [225]
Child et al. (1998) [226]
Corona et al. (2010) [227]
Paulsen et al. (2010b) [222]
Bloomer et al. (2004) [228]
Larsen et al. (2007) [229]
Sesto et al. (2005) [230]
Staples et al. (1999) [231]
Nosaka et al. (2002) [232]
Proske et al. (2004) [233]
Chapman et al. (2011b) [166]
Rodenburg et al. (1993) [234]
Nosaka et al. (2002b) [152]
Barash et al. (2004) [235]
Carter et al. (1994) [236]
Peters et al. (2003) [237]
Rodenburg et al. (1994) [15]
Cutlip et al. (2004) [238]
-4
-2
0
+2
+4
Favors secondary strength loss
Does not favor secondary strength loss

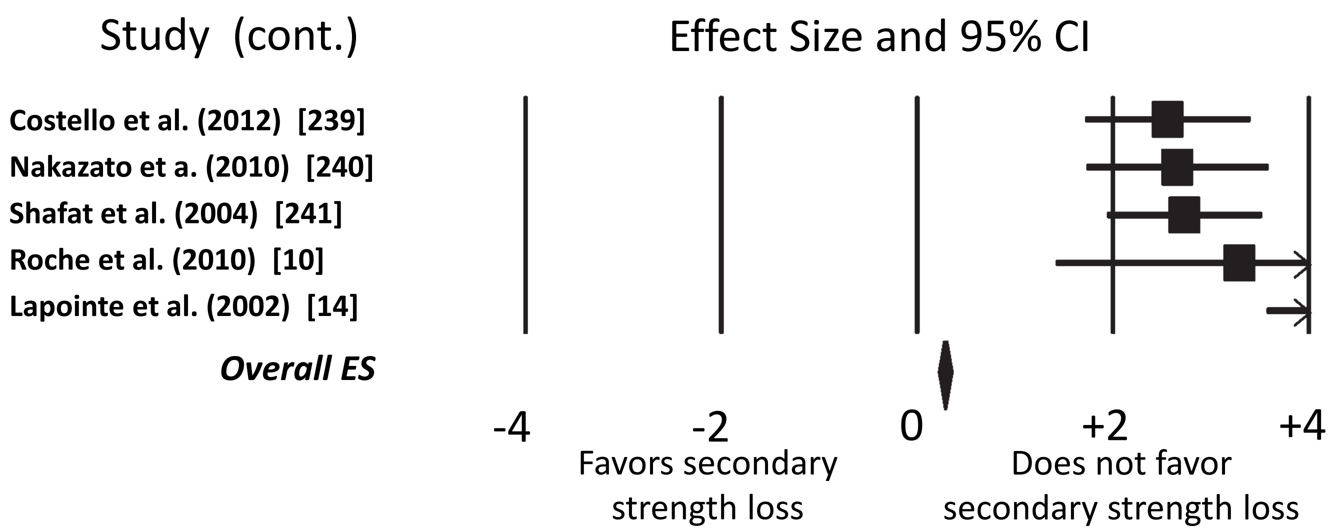

Electronic Supplementary Material Figure S1.
Forest plot of effect sizes (ES) from the 262 independent groups of subjects included in the overall meta-analysis. The center of a square represents the ES for a given study. The horizontal line running through a square represents the 95% confidence interval (CI) for the study ES. The weighting assigned to an independent group ES in the calculation of the overall ES is inversely related to its 95% CI. Studies are arranged from the lowest to highest ES. The diamond at the bottom represents the overall ES with the width of the diamond corresponding to the 95% CI for the overall ES. Lowercase letters after the publication year in the study listing indicate studies that had multiple independent groups of subjects.
